# Supplementary material for: The burden and correlates of bloodborne pathogens in a Zambian Blood Donor Cohort
Source: PLoS One. 2026 Apr 6;21(4):e0346680. doi: 10.1371/journal.pone.0346680 (PMC13052868; doi:10.1371/journal.pone.0346680)
Supplement: Attachment — Submitted filename: Response to Reviewers.docx [file pone.0346680.s004.docx]

**PONE-D-25-55464: The Burden and Correlates of Bloodborne Pathogens in a Zambian Blood Donor Cohort**

Journal Requirements:

When submitting your revision, we need you to address these additional requirements.

1. Please ensure that your manuscript meets PLOS ONE's style requirements, including those for file naming. The PLOS ONE style templates can be found at

<https://journals.plos.org/plosone/s/file?id=wjVg/PLOSOne_formatting_sample_main_body.pdf> and <https://journals.plos.org/plosone/s/file?id=ba62/PLOSOne_formatting_sample_title_authors_affiliations.pdf>

**Response**: We thank the editorial team for this comment. The manuscript has been formatted to the journal style requirements.

2. When completing the data availability statement of the submission form, you indicated that you will make your data available on acceptance. We strongly recommend all authors decide on a data sharing plan before acceptance, as the process can be lengthy and hold up publication timelines. Please note that, though access restrictions are acceptable now, your entire data will need to be made freely accessible if your manuscript is accepted for publication. This policy applies to all data except where public deposition would breach compliance with the protocol approved by your research ethics board. If you are unable to adhere to our open data policy, please kindly revise your statement to explain your reasoning and we will seek the editor's input on an exemption. Please be assured that, once you have provided your new statement, the assessment of your exemption will not hold up the peer review process.

**Response**: We thank the editorial team for this comment. The data availability statement has been revised. The raw data underlying the results presented in the study have been uploaded as a Supporting information file (S2).

3. Thank you for stating the following in the Competing Interests section:

[The authors have declared that no competing interests exist.].

We note that one or more of the authors are employed by a commercial company: Unilabs.

**Response**: We thank the editorial team for this comment. The Competing Interests section has been revised. The authors have read the journal's policy and have the following competing interests: Author [P.M] is a salaried employee of Unilabs. This does not alter our adherence to the journal's policies on sharing data and materials. The other authors have declared that no competing interests exist.

1. Please provide an amended Funding Statement declaring this commercial affiliation, as well as a statement regarding the Role of Funders in your study. If the funding organization did not play a role in the study design, data collection and analysis, decision to publish, or preparation of the manuscript and only provided financial support in the form of authors' salaries and/or research materials, please review your statements relating to the author contributions, and ensure you have specifically and accurately indicated the role(s) that these authors had in your study. You can update author roles in the Author Contributions section of the online submission form.

Please also include the following statement within your amended Funding Statement.

“The funder provided support in the form of salaries for authors [insert relevant initials], but did not have any additional role in the study design, data collection and analysis, decision to publish, or preparation of the manuscript. The specific roles of these authors are articulated in the ‘author contributions’ section.”

If your commercial affiliation did play a role in your study, please state and explain this role within your updated Funding Statement.

**Response**: We thank the editorial team for this comment. We have revised the funding statement. This research received no specific grant from any funding agency in the public, commercial, or not-for-profit sectors. The authors [P.M] is employed by Unilabs, but Unilabs provided no financial support specifically for this work. Moreover, we have verified the 'Author Contributions' section. The contributions of author [P.M] employed by Unilabs are articulated in terms of their direct scientific roles in this research such as data analysis, and manuscript drafting), confirming their involvement was academic in nature.

2. Please also provide an updated Competing Interests Statement declaring this commercial affiliation along with any other relevant declarations relating to employment, consultancy, patents, products in development, or marketed products, etc.

Within your Competing Interests Statement, please confirm that this commercial affiliation does not alter your adherence to all PLOS ONE policies on sharing data and materials by including the following statement: "This does not alter our adherence to PLOS ONE policies on sharing data and materials.” (as detailed online in our guide for authors <http://journals.plos.org/plosone/s/competing-interests>) . If this adherence statement is not accurate and there are restrictions on sharing of data and/or materials, please state these. Please note that we cannot proceed with consideration of your article until this information has been declared.

**Response**: We thank the editorial team for this comment. The Competing Interests section has been revised. The authors have read the journal's policy and have the following competing interests: Author [P.M] is a salaried employee of Unilabs. This does not alter our adherence to the journal's policies on sharing data and materials. The other authors have declared that no competing interests exist.

Please include both an updated Funding Statement and Competing Interests Statement in your cover letter. We will change the online submission form on your behalf.

**Response**: We thank the editorial team for this comment. We have revised the cover letter to include funding statement and competing interests

If the reviewer comments include a recommendation to cite specific previously published works, please review and evaluate these publications to determine whether they are relevant and should be cited. There is no requirement to cite these works unless the editor has indicated otherwise.

**Response**: We thank the editorial team for this comment.

Additional Editor Comments:

In addition to addressing the reviewers' comments, I strongly suggest analysing each factor and the titres of serological tests for each pathogen. An analysis limited to the educational status and blood grouping will bias the results.

**Response**: We sincerely thank the reviewer for these valuable insights. We have substantially revised our analysis in response to your recommendations:

- We have expanded our multivariable logistic regression model to include all variables in the dataset (age, sex, marital status, education, district, donor type, Rhesus factor, and ABO blood group). The revised analysis no longer focuses exclusively on education and blood group, thus avoiding the potential bias noted by the reviewer.
- We have conducted separate multivariable regression analyses for each specific transfusion-transmissible infection (HBV, HCV, HIV, and Syphilis) to identify whether different factors predict reactivity for different pathogens. These results are presented in a new supplementary table.
- We acknowledge the reviewer's suggestion regarding analysis of serological titers. Unfortunately, our laboratory's routine screening protocol uses qualitative (positive/negative) results for blood donor screening, and quantitative titer data were not systematically recorded in the donor registries. We have clarified this limitation in the manuscript.

Review Comments to the Author

Please use the space provided to explain your answers to the questions above. You may also include additional comments for the author, including concerns about dual publication, research ethics, or publication ethics. (Please upload your review as an attachment if it exceeds 20,000 characters)

Reviewer #1: The results of this study conducted in Zambia were as expected as recent high-quality studies and reports (systematic reviews, regional analyses, and large reports) from several African countries that identify the burden of bloodborne pathogens (particularly hepatitis B virus, hepatitis C virus, HIV, and syphilis/other bloodborne diseases like malaria) and examine their associations. Also, recent studies from Africa and other regions have examined whether ABO blood type (particularly blood type O) is associated with lower TTI risk. Multiple studies (from Sub-Saharan Africa, parts of Asia, and other low- and middle-income countries) find that lower education (below secondary) is associated with a higher prevalence of blood-borne infections among blood donors, while donors with secondary or higher education generally show lower infection rates — but there are important regional and methodological exceptions.

**Response**: We thank the reviewer for the kind feedback.

What's striking is that limitations of this study make it extremely is not reliable.

The study method was a cross-sectional study conducted using records, Data were obtained from routine blood donor records and screening registers. In regards to the level of education as a risk factor of transfusion-transmissible infections (TTIs), the author did not clarify whether there were using a structured/ standard blood transfusion service questionnaire for Socio-demographic characteristics and associated risk factors (educational level). If such a questionnaire is used in the study, it will be shown numbers of donors with primary and higher education and not just secondary. It is well known that education level is not a requirement for routine blood donation. Blood donation screening records refer to the process of using a register of previously deferred donors to check potential donors and prevent ineligible individuals from donating blood. Yes, the donor's educational level can be a factor in blood donation screening in some studies (a questionnaire is conducted beforehand for this purpose), although it is not used in the basic health screening process itself. The primary purpose of the screening is to ensure blood safety by assessing the donor's health. This is done through questionnaires and a confidential interview that checks their medical history, lifestyle, and physical health measures such as blood pressure, pulse, and hemoglobin levels.

**Response**: We thank the reviewer for this important critique on how data was collected. We have revised the methodology section to make this section clearer. It now reads “Data were extracted from routine blood donor screening registers. **It is important to note that these registers were designed for operational tracking and not for comprehensive socio-demographic analysis.** While fields for age, sex, donation type, and blood group were consistently completed, the fields for 'education level' and 'marital status' were **non-standardized and inconsistently recorded**, resulting in a high proportion of missing data ('not documented'). **These variables were not derived from a structured donor health assessment questionnaire**”.

Here few comments:

In Table 1. The median age of the cohort was 24 years, is this median age for infected and non infected (not mention in table), Could authors add the frequency of additional ages and corelate them to the level of education (secondary)?

0

**Response**: We thank the reviewer for this important suggestion and critique. We have revised table 1 to include age for the infected 24 years (19, 35) and non-infected 23 years (18, 33). We have also added frequency of additional ages. Moreover, while we agree that exploring the relationship between age and education would be informative, education data in our dataset were incompletely documented, with over 88% recorded as ‘not documented’ and only one education category (secondary) available. Performing a cross-tabulation or correlation analysis under these conditions would be statistically unreliable and potentially misleading. We have therefore retained education as a descriptive variable and clarified this limitation in Table 1 footnote, and Discussion.

Reviewer #2: This manuscript investigates the predictors of the infected blood donors with four pathogens. In fact, the authors analysed only two factors; the educational status and the blood groups. The authors missed to analyse many factors that listed in the table 2. In addition, the presented data are categorized rather than continuous which described rather than to analyzes the results Accordingly, the presented results will not free from bias.

Using appropriate statistical analysis will improve the quality of the study

**Response**: We thank the reviewer for this important suggestion and critique. We have reanalyzed and revised table 2 and have now included all variables from the dataset in a comprehensive multivariable logistic regression model, as presented in the revised Table 2. No predictors were excluded from the analysis. Secondly, the changes made using the full model minimizes unnecessary categorization, directly addressing the potential for omitted variable bias and information loss, strengthening the validity of our findings.

The titles of the serological tests need to be added and analyzed

**Response**: We thank the reviewer for this important suggestion. We have revised the method section to make the serological tests clearer. We have also revised the figure which was meant to illustrate pathogens analyzed by serological test.

The figures are not informative, and needs more clarifications and improvements.

**Response**: We thank the reviewer for this important suggestion and critique. We have revised the figure 1 and have converted figure 2 to table 3 to make them clearer and informative.
