# Supplementary material for: The burden and correlates of bloodborne pathogens in a Zambian Blood Donor Cohort
Source: PLoS One. 2026 Apr 6;21(4):e0346680. doi: 10.1371/journal.pone.0346680 (PMC13052868; doi:10.1371/journal.pone.0346680)
Supplement: Attachment — Submitted filename: Response_to_Reviewers_auresp_2.docx [file pone.0346680.s005.docx]

**Point by Point Response to reviewer comments**

Review Comments to the Author

Please use the space provided to explain your answers to the questions above. You may also include additional comments for the author, including concerns about dual publication, research ethics, or publication ethics. (Please upload your review as an attachment if it exceeds 20,000 characters)

Reviewer #1: The author to some extent has responded to the comments, It's a decent effort , though it lacks depth. Just here one comment, in tables 1, 2, age group 14-24 years. Does 14 years eligible for blood donation in Zambia?!

Usually Standard age of donation is 18 to 65 years old, as organizations focus on donors who are in good health, meet weight standards, hemoglobin standards, and have a stable medical history to ensure donor safety and blood quality.

The minimum age is usually 16 or 17 with parental consent in some places. But at 14 it is restricted as individuals are still developing, and to protect their health.

**Response**

We thank the reviewer for the insight comment and feedback. We agree with the reviewer’s comment. The minimum age permitted to donate blood in Zambia and in our study was 16 years of age. We sincerely apologies as this was a typographic error. We have revised tables 1 and 2, age group from 14-24 years to 16-24 years.

Reviewer #3: This cross sectional study present the burden and correlates of bloodborne pathogens among blood donors in a Zambian cohort, particular focus on identifying demographic, behavioral, and operational factors associated with transfusion-transmitted infections (TTI) prevalence. However, my concerns/suggestions are not necessarily a reflection of the quality of the work, as follows:

•Overall, the introduction would benefit from a clearer and more cohesive rationale, with smoother transitions between paragraphs. In particular, the discussion of cancer treatment implications lacks a sufficiently developed biological or clinical rationale. Strengthening the mechanistic or pathophysiological basis for this link would help justify the study’s relevance and potential impact.

**Response**

We thank the reviewer on this insight comment. We have revised the paragraphs to improve clinical rationality and to make logical flow. We have also removed cancer treatment from the introduction.

•While the reported TTI prevalence (25.7%) is contextualized through comparison with prior studies, the inclusion of Saudi Arabia as a comparator (Ref. 16) in the discussion is epidemiologically inappropriate and potentially misleading. Saudi Arabia represents a distinct donor population with markedly different baseline infection prevalence, donor recruitment strategies, and screening protocols compared with Sub-Saharan African settings. Comparative interpretations should therefore be limited to regions with broadly comparable epidemiological and healthcare contexts.

**Response**

We thank the reviewer on this insight comment. We have revised and removed the Ref. 16

•Some comments about figures and tables here and there!

oIn Figure 1:

The flow diagram oversimplifies cohort selection and does not adequately describe exclusion steps or eligibility criteria, limiting reproducibility and interpretability.

The figure lacks a descriptive title and legend, and visual elements appear decorative rather than informative.

**Response**

We thank the reviewer on this insight comment. We have revised and removed the figure.

oIn Table 1:

The high proportion of undocumented variables severely limits interpretability and undermines the validity of comparisons.

**Response**

We thank the reviewer on this insight comment. We have revised and removed undocumented category. We have mentioned this as a limitation.

Testing both continuous and categorized age variables introduces redundancy and inflates the number of statistical comparisons.

**Response**

We thank the reviewer on this insight comment. We have revised and removed continuous variable for age.

Blood group frequencies differ substantially across populations and may be confounded by ethnicity, geography, or donor selection. It is highly recommended to clearly state that this table is univariate.

**Response**

We thank the reviewer on this insight comment. We have revised and replace bivariate to univariate.

Several comparisons involve extremely small cell counts, violating assumptions of chi-square testing.

**Response**

We thank the reviewer on this insight comment. We have added fishers exact test for small cel counts. The statement now reads “Chi-square tests or fishers exact test for small cel counts was used for categorical variables”.

•In summary, while the study addresses an important question and is based on a valuable dataset, substantial revisions are needed to improve conceptual coherence, epidemiological interpretation, methodological transparency, and biological depth. Addressing the points above would significantly enhance the rigor, clarity, and overall impact of the manuscript.

**Response**

We thank the reviewer on this insight comment. We have revised the entire manuscript attending to the reviewers’ comments.

Reviewer #4: Dear Authors

Thank you for submitting your research for publication. The manuscript addresses an important topic. However, several methodological, conceptual, and reporting issues must be addressed before the manuscript can be considered for publication. Major revisions are required to ensure scientific rigor, clarity, and alignment between study aims, analysis, and conclusions.

Major revision issues:

1. There is a mismatch between stated study aims and analyses conducted. The stated study aim includes addressing the reasons behind high blood discard rates beyond TTIs; however, the analyses presented focus exclusively on transfusion-transmissible infections. Non-TTI causes of discard (e.g., expiry, inadequate volume, storage or handling issues) are not investigated.

** Recommendation: Revise the study aim to align with the analyses conducted or expand the analysis to include non-TTI discard reasons. Adjust conclusions accordingly.

**Response**

We thank the reviewer for the insightful comment provided. We have revised the aim of the study to suite the investigation conducted. The aim of the study now reads “*This study aims to determine the burden and correlates of transfusion-transmissible infections among blood donors in a Zambian cohort, with particular focus on identifying demographic and donor-related factors associated with TTI prevalence. By elucidating these factors, the study seeks to inform strategies for improving blood safety and reducing TTI-related discards in the region.”*

2. The conclusion that individuals with secondary education have a higher risk of infection compared to those with “not documented education level” is not methodologically justified. The “not documented” category does not represent a valid comparison group and raises concerns regarding misclassification and missing data bias. Authors should justify the inclusion of this category as a reference group or reanalyse the data by treating undocumented education as missing. Comparative analyses should be restricted to clearly defined education levels. The authors should refrain from making risk-based inferences using this category and revise both the analysis and conclusions.

** Recommendation: Remove or reanalyse education-level comparisons, treating undocumented education as missing data. Condense or remove the related discussion and reframe it as a limitation.

**Response**

We thank the reviewer on this insight comment. We have revised the manuscript by treating education undocumented education as missing. We have further removed this from the discussion section and have added it as a limitation.

3. Limited screening infrastructure would be expected to result in underestimation, not higher observed prevalence as stated in lines 68-71.

** Recommendation: Clarify the mechanism or revise wording to avoid causal inconsistency between detection capacity and observed prevalence.

**Response**

We thank the reviewer on this insight comment. We have revised the manuscript to avoid a causal inconsistency.

Detailed comments/recommendations:

Abstract:

Line 46: “further mechanistic investigation...”

** Rephrase or explain “mechanistic investigation”

**Response**

We thank the reviewer on this insight comment. We have revised the phrase and replaced it with “suggesting a potential biological basis that requires further study”.

Introduction:

Line 50-51: “Blood transfusion remains a critical... that is essential for treating various conditions including surgical procedures, trauma management…”.

** This statement is imprecise. Blood transfusion is a supportive, life-saving intervention used when clinically indicated (e.g., perioperative haemorrhage or trauma-related blood loss), rather than a treatment for surgery itself. The authors are encouraged to revise this sentence for clinical accuracy and clarity.

**Response**

We thank the reviewer on this insight comment. We have revised the phrase for clinical accuracy and clarity.

Line 68-71: “These rates are substantially higher than those observed in high-income countries, reflecting the complex interplay of factors including high community prevalence of these infections, reliance on replacement and first-time donors, and limitations in screening infrastructure”.

** The attribution of higher observed prevalence to “limitations in screening infrastructure” is conceptually unclear. Limited screening capacity would be expected to result in underestimation rather than higher measured prevalence. This contradiction should be addressed or the wording revised.

**Response**

We thank the reviewer on this insight comment. We have revised the phrase to resolve the contradiction in the wording.

Line 83-85: “Furthermore, the economic impact of discarded units, combined with the costs of screening and confirmatory testing, places additional strain on already resource-constrained blood transfusion service”.

** This statement, although might be correct, does not directly relate to the study aim or contribute to the interpretation of the results. Its inclusion appears tangential and should be justified or omitted.

**Response**

We thank the reviewer on this insight comment. We have revised and omitted the phrase.

Line 88-89: “This study aims to address the reasons behind the high discard rates and explore the factors contributing to the prevalence of TTIs in the province”.

** The analyses presented focus exclusively on transfusion-transmissible infections. Non-TTI causes of discard are not investigated. The authors should either revise the study aim to align with the analyses conducted or expand the analysis to include non-TTI discard reasons. Adjust conclusions accordingly.

**Response**

We thank the reviewer on this insight comment. We have revised the study aim to align with the analyses conducted.

Result:

Line 149: “A total of 2,667 participants were included in the analysis, of whom 686 (25.7%) had screened positive for infected blood.”

** Rephrase statement for clarity and accuracy.

**Response**

We thank the reviewer on this insight comment. We have revised the statement for clarity and accuracy.

Line 153-154: “Participants with a secondary education had a significantly higher infection prevalence (31.5%) compared to those whose education level was not documented (25.0%; p=0.012).”.

** As mentioned above, authors should justify the inclusion of “not documented education level” category as a reference group or reanalyse the data by treating undocumented education as missing. Comparative analyses should be restricted to clearly defined education levels.

**Response**

We thank the reviewer on this insight comment. We have excluded undocumented cases from comparative analyses. We have also added footnote for clarity.

Discussion:

Line 210-222:

** The extended discussion of education level relies on a comparison with the “not documented” category, which the authors themselves acknowledge represents missing and heterogeneous data. As such, inferential comparisons and adjusted analyses using this category are not valid. The discussion remains speculative and introduces unsupported claims regarding the risk profile of individuals with missing education data. I recommend removing this paragraph and instead briefly noting the issue of missing educational data as a limitation.

**Response**

We thank the reviewer on this insight comment. We have removed this paragraph from

The discussion and instead have replace with missing education data as a limitation

Limitations:

** The limitations should be expanded on to include a more discussion of the potential confounders.

**Response**

We thank the reviewer on this insight comment. We have expanded on limitations
