# Supplementary material for: The burden and correlates of bloodborne pathogens in a Zambian Blood Donor Cohort
Source: PLoS One. 2026 Apr 6;21(4):e0346680. doi: 10.1371/journal.pone.0346680 (PMC13052868; doi:10.1371/journal.pone.0346680)
Supplement: Attachment — Submitted filename: Response_to_Reviewers_auresp_3.docx [file pone.0346680.s006.docx]

**Point by Point responses to Reviewers’ comments**

We thank the reviewers for their helpful comments. The manuscript has been revised to address their feedback, as outlined in our responses.

Reviewer #3: Thank you for the revised manuscript. The revision shows clear improvement and addresses several earlier concerns; however, some important issues remain. In particular, the handling of the “undocumented” education variable is inconsistent, as it continues to appear in adjusted analyses and interpretations even though it is described as excluded. In addition, there are residual internal inconsistencies, including an incorrect age category and incomplete alignment between the Methods, Tables, Results, and Abstract. Addressing these points in a focused revision will be necessary to ensure methodological clarity and internal consistency.

**Response**
We thank the reviewers for their insightful feedback and observations. We have removed ‘undocumented education’ from all inferential analyses and clarified that it represents missing data. All age categories are now consistent, and the Abstract has been aligned with the final Results. We thank the reviewers for their insightful comments, which have strengthened the manuscript.

Reviewer #4: Dear Authors,

We all agree that "Undocumented education" is not a true educational category.

The inclusion of “undocumented education level” as a predictor in univariable analysis remains methodologically inappropriate. This category represents missing data rather than a true exposure, and odds ratios derived from it are not interpretable, regardless of statistical significance. The reported association likely reflects patterns in data completeness rather than a meaningful relationship with infection risk. I strongly recommend removing this variable from inferential analyses and treating missing education as a limitation rather than a risk factor.

**Response**
We thank the reviewers for their insightful feedback and observations. We have revised and reviewed the manuscript. We have corrected as suggested.

There is a discrepancy between the abstract and the Results section. The authors should reconcile these differences, clarify whether the reported estimate is from univariable or multivariable analysis, and ensure that the abstract accurately reflects the final results presented in the manuscript.

**Response**

We thank the reviewers for their insightful feedback and observations. We have revised and have clearly indicated that the multivariable analysis was conducted.

In addition, given the methodological concerns regarding the interpretability of the “undocumented education level” category, the authors should carefully reconsider whether this variable should be highlighted in the abstract at all.

**Response**

We thank the reviewers for their insightful feedback. We have revised the abstract and have ensured that education status is not appearing. We have further omitted education status in multivariable analysis. But we have shown education under descriptive as percentages in table 1 participant characteristics. We have also updated the method section to add a statement of no inclusion of education status.
